# India’s ban on antimicrobial fixed-dose combinations: winning the battle, losing the war?

**DOI:** 10.1186/s40545-022-00428-w

**Published:** 2022-04-28

**Authors:** Giorgia Sulis, Richeek Pradhan, Anita Kotwani, Sumanth Gandra

**Affiliations:** 1 Department of Epidemiology, Biostatistics and Occupational Health, McGill University, Montreal, QC Canada; 2 Department of Pharmacology, V. P. Chest Institute, University of Delhi, Delhi, India; 3 Division of Infectious Diseases, Department of Medicine, Washington University School of Medicine in Saint Louis, Saint Louis, MO USA

**Keywords:** India, Antimicrobials, Antimicrobial resistance, Fixed-dose combinations, Pharmaceutical policy, AWaRe framework

## Abstract

**Background and objectives:**

India, the country with the largest market availability of antimicrobial fixed-dose combinations (FDCs), banned certain antimicrobial FDCs in September 2018. Our objective was to examine the impact of Government ban on the sales of antimicrobial FDCs.

**Methods:**

The sales patterns of 14 of the 26 banned antimicrobial FDCs were analyzed using monthly private sector drug sales data from IQVIA (a comprehensive and nationally representative drug sales database) between January 2018 and December 2019. We carried out descriptive analyses to evaluate the trend in sales over time for banned and non-banned antimicrobial FDCs using cumulative sales volumes.

**Results:**

Overall, the cumulative sales volume of banned antimicrobial FDCs declined by 75% between January and September 2018 and the same months of 2019, although some banned FDCs continued to be available in significant volumes. The effectiveness of the ban was offset by several pathways. First, the sales of combinations containing moieties belonging to the same drug-classes as the antimicrobials in the banned FDCs increased after the ban. Second, while certain formulations of particular combinations were banned, the sales of other non-banned formulation of these combinations increased. Third, in some cases, products containing new non-antimicrobial components added to the banned combinations remained available.

**Interpretation and conclusions:**

While sales of the banned antimicrobial FDCs decreased in 2019, we identified several mechanisms that counterbalanced the ban, including implementation failure, rising sales of congeners, and products with additional non-antimicrobial components.

**Supplementary Information:**

The online version contains supplementary material available at 10.1186/s40545-022-00428-w.

## Introduction

India is the top consumer of antimicrobials worldwide and bears among the highest burden of antimicrobial resistance (AMR) [1, 2]. A particular challenge in AMR control in India is the highest market availability of fixed-dose combinations (FDCs) of antimicrobials in the world [3]. FDCs are formulations that combine two or more active ingredients in fixed ratios in a single dosage form. Reasons behind the popularity of FDCs of antimicrobials include the better patient adherence, lower costs, and wider antimicrobial coverage amid potential uncertainty about causative microorganisms [4, 5]. Although, antimicrobial FDCs have been critical in improving clinical outcomes among patients with certain infections such as tuberculosis and human immunodeficiency virus (HIV), the use of such FDCs for routine bacterial infections is inappropriate as it drives AMR by selecting co-resistant microorganisms [6, 7]. Thus, their indiscriminate use is widely discouraged, including in the World Health Organization’s AWaRe (Access, Watch & Reserve) framework of antimicrobial prescribing [8].

Many FDCs marketed in India have never been approved by the country’s Central Drugs Standard Control Organization (CDSCO), their approval owed to state-level regulatory bodies that, at times, may lack sufficient technical expertise to make such decisions [9]. In fact, after a decade-long legal battle with the pharmaceutical companies, the Government banned 26 antimicrobial FDCs in September 2018 [10]. However, the banned FDCs represent a small proportion of all antimicrobial FDCs available in the Indian market [3, 9]. There are at least 43 systemic antimicrobial FDCs that are still available in the Indian market that are considered irrational [11]. So, there is a possibility of increase in utilization of non-banned antimicrobial FDCs to replace banned FDCs compromising the impact of the ban. The objective of this study is to examine the impact of the Government ban on the utilization of select antimicrobial FDCs in India. Accordingly, we used a comprehensive and nationally representative drug sales database to examine the sales patterns of antimicrobial FDCs before and after September 2018.

## Materials and methods

We used monthly drug sales data from IQVIA Inc., a private company that collects data on several healthcare-related indicators across countries and is considered as a reliable source of antimicrobial sales data which has been previously used in many studies [3, 12–14]. IQVIA covers 95% of the private market in India, including both outpatient and inpatient use of antimicrobials, and capturing both generic and brand names of each drug product. The data in the present study were collected between January 2018 and December 2019 and allowed to examine sales patterns of 14 of the 26 banned antimicrobial FDCs. The analysis was restricted to only 14 banned antimicrobial FDCs as data were not available for other 12 banned antimicrobial FDCs in the IQVIA dataset. Sales volumes used for our analyses were expressed in standard units (SU), where 1 SU (corresponding to one dose) was defined as a single tablet, capsule, ampoule, vial, or a 5 mL liquid preparation for oral consumption, in line with previously conducted studies [12, 15]. Descriptive analyses were carried out to evaluate the change in sales over time for banned and non-banned antimicrobial FDCs available in the IQVIA database using cumulative sales volumes and proportions as appropriate. FDCs that were available in India in 2018 are listed in Additional file 1: Appendix Table S1. To examine the effect of the ban while accounting for seasonal trends, we compared sales data from the same months (January to September) of 2018 and 2019. Percentage change in sales was calculated as:

$$\frac{(\mathrm{Cumulative}\, \mathrm{sales}\, \mathrm{of}\, \mathrm{a}\, \mathrm{product}\, \mathrm{in}\, \mathrm{Jan}{-}\mathrm{Sep}\, 2018)-(\mathrm{Cumulative}\, \mathrm{sales}\, \mathrm{of}\, \mathrm{that}\, \mathrm{product}\, \mathrm{in}\, \mathrm{Jan}{-}\mathrm{Sep}\, 2019)}{\mathrm{Cumulative}\, \mathrm{sales}\, \mathrm{of}\, \mathrm{that}\, \mathrm{product}\, \mathrm{in}\, \mathrm{Jan}{-}\mathrm{Sep}\, 2018}\times 100.$$

Because no identifiable information about living individuals were obtained, this study was exempted from ethics review.

## Results and discussion

**Fig. 1:**
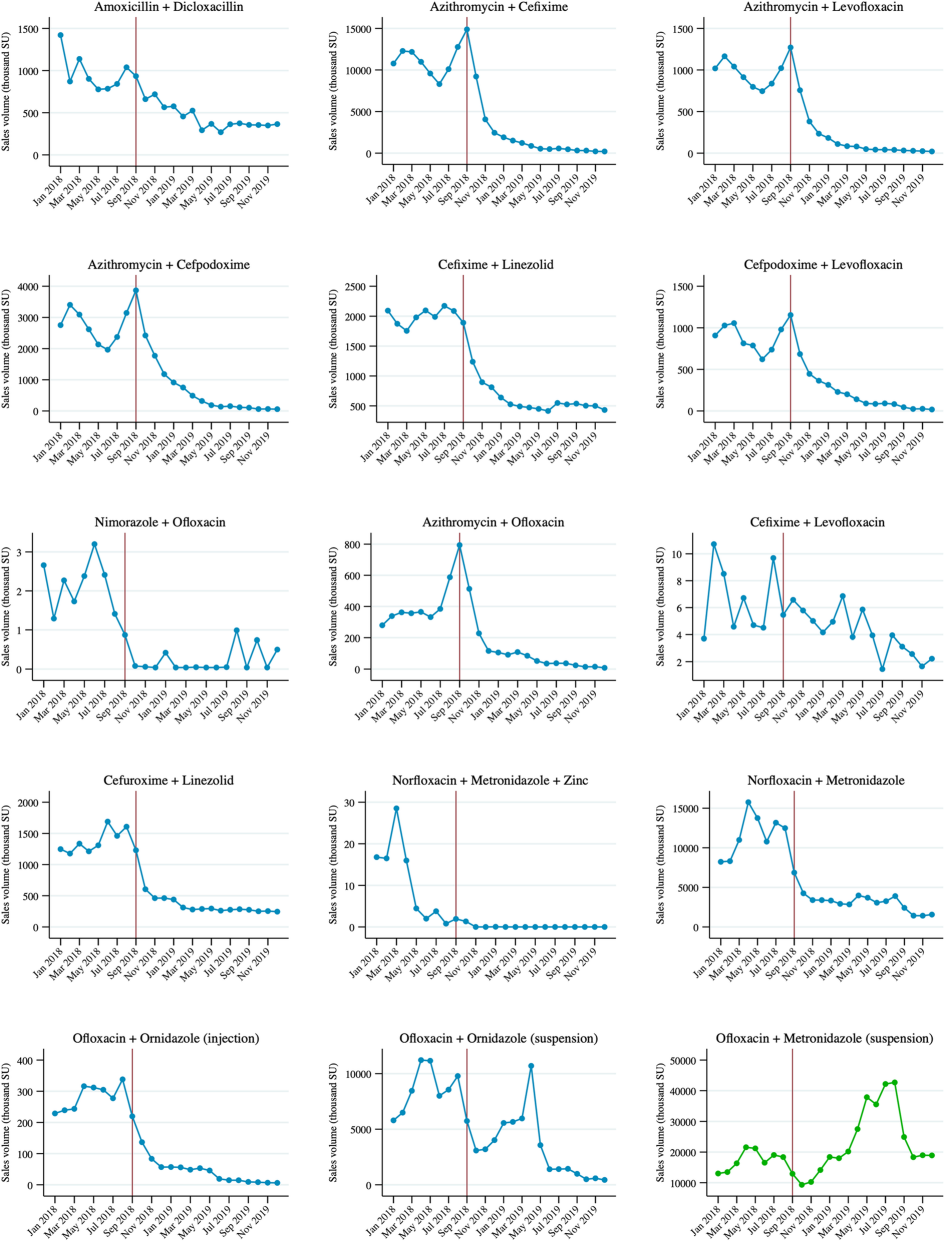
Trends in sales of banned antimicrobial fixed-dose combination (FDC) formulations between January 2018 and December 2019. The graph is based on monthly sales volumes of each product (across brands) expressed in standard units (i.e., doses) as obtained from IQVIA Inc. The ban on select antimicrobial FDCs was introduced in India in September 2018. *Note:* Ofloxacin+metronidazole suspension was not banned and is presented only for comparison with banned formulations of ofloxacin+ornidazole suspension

Overall, the cumulative sales volume of banned antimicrobial FDCs declined by 75% between January and September 2018 (i.e., before the ban) and the same months of 2019, from 365 million SUs in 2018 to 91 million SUs in 2019. However, closer scrutiny helps illuminate aspects that need greater attention to tackle the problem of antimicrobial FDCs at large. For example, some banned FDCs continued to be available in significant volumes in 2019 (e.g., norfloxacin+metronidazole, amoxicillin+dicloxacillin, cefixime+linezolid, and cefuroxime+linezolid, Fig. 1). This indicates the need for stricter implementation of the regulatory decision. Sale patterns of FDC formulations that had not been banned, however, showed how the effectiveness of the regulation might had been offset by several bypassing pathways. First, the sales of combinations containing moieties belonging to the same drug-classes as the antimicrobials in the banned FDCs increased after the ban. For example, although sales of banned ofloxacin+ornidazole suspension decreased by 51% in 2019 (75 million SUs in 2018 vs 37 million SUs in 2019), sales of ofloxacin+ metronidazole suspensions increased by 80% (15 million SUs in 2018 vs 27 million SUs in 2019) (Fig. 1). This could potentially be due to an increased demand for products that met the same needs as the banned FDCs. Second, although ofloxacin+ ornidazole injection and suspension formulations were banned, ofloxacin+ornidazole tablets were not banned. Interestingly, the non-banned ofloxacin+ornidazole tablet FDC is among the most sold antimicrobial FDCs in India and, according to our data, its sales increased by 6% in 2019 (344 million SUs in 2018 vs. 364 million SUs in 2019). Third, although sales of the specific FDCs banned declined, products containing the same two antimicrobials along with one or more non-antimicrobial components remained available. For example, while sales of azithromycin+cefixime or norfloxacin+metronidazole diminished, that of azithromycin+cefixime+Lactobacillus or norfloxacin+metronidazole+Bacillus coagulans, that had not been banned, persisted (Additional file 1: Appendix Table S2). Similarly, while levofloxacin+ornidazole+alpha tocopherol acetate and ofloxacin+ornidazole+zinc bisglycinate were banned, levofloxacin+ornidazole or ofloxacin+ornidazole without additional components were not included in the Government order. Thus, because the ban had been instituted on only a few of the FDCs available, and because of the above-described implementation failure or bypassing mechanisms, there was little effect on the sales of all discouraged FDCs marketed in India, with a mere 8% reduction between January and September of 2018 and 2019 (2467 million SUs in 2018 vs 2265 million SUs in 2019).

Given that India has the highest number of antimicrobial FDCs available in the market [16], an FDC ban can both impact the antimicrobial consumption pattern within the country and can serve as a template for FDC control elsewhere. However, this analysis reveals that the task of restricting the use of antimicrobial FDCs is a complex one. At the health-systems level, reasons as to why the FDCs have come to be the preferred prescribing choices must be addressed [16]. Uncertainty regarding the causative microorganism that might make concurrent use of antimicrobials preferred should be countered by developing and expanding cheap and accurate point-of-care antimicrobial diagnostics [17, 18]. Similarly, healthcare providers should be regularly updated on the common bacterial pathogens causing prevalent infections in the facilities they serve and their antimicrobial susceptibility patterns [19, 20]. In addition, the ethics of promotional practices of pharmaceutical representatives based on incomplete medical information and their impact on FDC prescribing should be investigated [21]. At the regulatory level, while designing new prohibitions, consideration should be given to the above-described pathways by which such prohibitions can be bypassed. Furthermore, given that little published evidence exists on the superiority of FDCs [11, 22, 23], clear explanations should be provided as to why only some FDCs were banned and not others. Finally, given the market dominance of FDCs in many jurisdictions, the possibility that abrupt banning may restrict access to even the component drugs should be considered. Instead, a planned phasing out might be deemed more appropriate in such scenarios.

Our study has some strengths. It is the first analysis examining the effect of the landmark FDC ban in India in 2018. Our results also uncover hitherto unstudied mechanisms by which the regulations have been bypassed. Because of the wide coverage of IQVIA database, our study accounts for both over the counter and prescription-based antimicrobial sales throughout the country. Our study also has some limitations. Although our analysis was based on ecological data, pharmaceutical sales data are highly correlated with patient-level consumption and are considered a good indicator of antimicrobial use in the community [1]. Moreover, the dataset did not include information on public sector antimicrobial consumption. However, it should be noted that, in the public sector, pharmaceutical procurement is mostly based on the National Essential Medicine List (EML) or State EML, which do not include discouraged FDCs. Given that the private sector consumption is the dominant mode of antimicrobial use in India, our analysis represents the majority of the antimicrobial consumption occurring nationwide before and after the ban, which further highlights the relevance of our study.

In conclusion, while the reductions in sales of the banned FDCs show that legal actions can be partially successful, we observed an increase in sales of non-banned antimicrobial FDCs. Therefore, effective control of antimicrobial FDC consumption will need more carefully crafted regulatory and societal solutions.

## Supplementary Information

**Additional file 1: Table S1.** List of 76 fixed dose combinations (FDCs) in the IQVIA database, of which 63 are categorized as “Discouraged” as per the AWaRe framework by the World Health Organization. **Table S2.** Sales volumes of banned and non-banned formulations of antimicrobial fixed dose combinations (FDCs) in India before and after the ban on FDCs.

## Data Availability

Data cannot be shared publicly because of license agreement with the IQVIA Inc. The data underlying the results presented in the study are available from IQVIA Consulting and Information Services India Pvt. Ltd. https://www.iqvia.com/locations/india.
