# Supplementary material for: India’s ban on antimicrobial fixed-dose combinations: winning the battle, losing the war?
Source: J Pharm Policy Pract. 2022 Apr 28;15:33. doi: 10.1186/s40545-022-00428-w (PMC9047325; doi:10.1186/s40545-022-00428-w)
Supplement: Supplementary file 1 — Additional file 1: Table S1. List of 76 fixed dose combinations (FDCs) in the IQVIA database, of which 63 are categorized as “Discouraged” as per the AWaRe framework by the World Health Organization. Table S2. Sales volumes of banned and non-banned formulations of antimicrobial fixed dose combinations (FDCs) in India before and after the ban on FDCs. [file 40545_2022_428_MOESM1_ESM.pdf]

Table S1. List of 76 fixed dose combinations (FDCs) in the IQVIA database, of which 63 are categorized as "Discouraged" as per the AWaRe framework by the World Health Organization. Fourteen of these FDCs were banned in India in September 2018.

Antibiotic combination	AWARE category	Included in ban	Notes
Amoxicillin/Dicloxacillin	Discouraged	YES	Only combinations with Serratiopeptidase or without additional components are currently banned, but products containing *Lactobacillus*, *Bacillus coagulans* or *Saccharomyces* are also on the market.
Amoxicillin/Tinidazole	Not yet included	YES	Only combinations without additional components are currently banned, but products containing omeprazole are on the market.
Azithromycin/Cefixime	Discouraged	YES	Only combinations without additional components are currently banned, but products containing *Lactobacillus*, *Bacillus coagulans* or lactic acid are also on the market.
Azithromycin/Cefpodoxime	Discouraged	YES	Only combinations without additional components are currently banned, but products containing *Lactobacillus* are also on the market.
Azithromycin/Levofloxacin	Discouraged	YES	Only combinations without additional components are currently banned, but products containing *Lactobacillus* are also on the market.
Levofloxacin/Ornidazole	Discouraged	YES	Only combinations with alpha-tocopherol acetate are currently banned, but products without it are on the market.
Norfloxacin/Metronidazole	Discouraged	YES	Only combinations without additional components or with zinc acetate are currently banned, but products containing *Bacillus coagulans* or simeticone are also on the market.
Ofloxacin/Metronidazole	Not yet included	YES	Only combinations with zinc acetate are currently banned, but products without zinc and with racecadotril or simeticone are on the market.

Antibiotic combination	AWARE category	Included in ban	Notes
Ofloxacin/Ornidazole	Discouraged	YES	Oral suspensions and injections are banned if without additional components. Tablet formulations are banned only if combined with zinc bisglycinate, but products with additional components are also on the market.
Azithromycin/Ofloxacin	Discouraged	YES	
Cefixime/Levofloxacin	Discouraged	YES	
Cefixime/Linezolid	Discouraged	YES	
Cefpodoxime/Levofloxacin	Discouraged	YES	
Cefuroxime/Linezolid	Discouraged	YES	
Nimorazole/Ofloxacin	Discouraged	YES	
Amikacin/Cefepime	Discouraged	NO	
Amoxicillin/Cloxacillin	Discouraged	NO	
Amoxicillin/Flucloxacillin	Discouraged	NO	
Amoxicillin/Sulbactam	Discouraged	NO	
Ampicillin/Cloxacillin	Discouraged	NO	
Ampicillin/Dicloxacillin	Discouraged	NO	
Ampicillin/Flucloxacillin	Discouraged	NO	
Azithromycin/Fluconazole/ Ornidazole	Not yet included	NO	
Cefadroxil/Clavulanate	Discouraged	NO	
Cefalexin/Clavulanate	Discouraged	NO	
Cefepime/Sulbactam	Discouraged	NO	
Cefepime/Tazobactam	Discouraged	NO	
Cefixime/Cefpodoxime	Discouraged	NO	
Cefixime/Clavulanate	Discouraged	NO	
Cefixime/Cloxacillin	Discouraged	NO	
Cefixime/Dicloxacillin	Discouraged	NO	
Cefixime/Moxifloxacin	Discouraged	NO	
Cefixime/Ofloxacin	Discouraged	NO	
Cefixime/Ornidazole	Discouraged	NO	
Cefixime/Sulbactam	Discouraged	NO	
Cefoperazone/Sulbactam	Discouraged	NO	
Cefoperazone/Tazobactam	Discouraged	NO	
Cefotaxime/Sulbactam	Discouraged	NO	
Cefpirome/Sulbactam	Discouraged	NO	
Cefpodoxime/Clavulanate	Discouraged	NO	

Antibiotic combination	AWARE category	Included in ban	Notes
Cefpodoxime/Cloxacillin	Discouraged	NO	
Cefpodoxime/Dicloxacillin	Discouraged	NO	
Cefpodoxime/Ofloxacin	Discouraged	NO	
Cefpodoxime/Sulbactam	Discouraged	NO	
Ceftazidime/Sulbactam	Discouraged	NO	
Ceftazidime/Tazobactam	Discouraged	NO	
Ceftazidime/Tobramycin	Discouraged	NO	
Ceftizoxime/Sulbactam	Discouraged	NO	
Ceftizoxime/Tazobactam	Discouraged	NO	
Ceftriaxone/Sulbactam	Discouraged	NO	
Ceftriaxone/Tazobactam	Discouraged	NO	
Ceftriaxone/Vancomycin	Discouraged	NO	
Cefuroxime/Clavulanate	Discouraged	NO	
Cefuroxime/Ornidazole	Not yet included	NO	
Cefuroxime/Sulbactam	Discouraged	NO	
Ciprofloxacin/Metronidazole	Discouraged	NO	
Ciprofloxacin/Ornidazole	Discouraged	NO	
Ciprofloxacin/Tinidazole	Discouraged	NO	
Clarithromycin/Tinidazole	Not yet included	NO	
Diloxanide/Metronidazole	Not yet included	NO	
Doxycycline/Ornidazole	Not yet included	NO	
Doxycycline/Tinidazole	Discouraged	NO	
Fluconazole/Ornidazole	Not yet included	NO	
Fluconazole/Tinidazole	Not yet included	NO	
Furazolidone/Metronidazole	Not yet included	NO	
Gatifloxacin/Ornidazole	Discouraged	NO	
Levofloxacin/Metronidazole	Discouraged	NO	
Meropenem/Sulbactam	Discouraged	NO	
Meropenem/Tazobactam	Discouraged	NO	
Metronidazole/Nalidixic acid	Not yet included	NO	
Norfloxacin/Tinidazole	Discouraged	NO	
Ofloxacin/Tinidazole	Discouraged	NO	
Penicillin G/Streptomycin	Discouraged	NO	
Tetracycline/Tinidazole	Not yet included	NO	
Ticarcillin/Clavulanate	Discouraged	NO	

Table S2. Sales volumes of banned and non-banned formulations of antimicrobial fixed dose combinations (FDCs) in India before and after the ban on FDCs. Data were obtained from IQVIA Inc. Banned and non-banned formulations differ in terms of additional non-antimicrobial components.

Fixed dose combination	Cumulative sales volume in thousand standard units			
	Banned formulations		Non-banned formulations	
	Jan-Sep 2018	Jan-Sep 2019	Jan-Sep 2018	Jan-Sep 2019
Amoxicillin/Dicloxacillin	8709	3583	14498	12272
Azithromycin/Cefixime	101873	7880	957	1148
Azithromycin/Levofloxacin	8811	659	0	1
Azithromycin/Cefpodoxime	25353	3183	3	0
Norfloxacin/Metronidazole	100352	29407	61	82
Ofloxacin/Ornidazole	77709	37021	343974	364426
